# Tumor necrosis factor alpha has an early protective effect on retinal ganglion cells after optic nerve crush

**DOI:** 10.1186/s12974-014-0194-3

**Published:** 2014-11-19

**Authors:** Caitlin E Mac Nair, Kimberly A Fernandes, Cassandra L Schlamp, Richard T Libby, Robert W Nickells

**Affiliations:** Department of Ophthalmology and Visual Sciences, University of Wisconsin, 571A MSC – 1300 University Ave., Madison, WI 53706 USA; Cellular and Molecular Pathology Graduate Program, University of Wisconsin, 3170-10 K/L MFCB – 1685 Highland Ave., Madison, WI 53705 USA; Flaum Eye Institute, University of Rochester Medical Center, 601 Elmwood Ave., Box 659, Rochester, NY 14642 USA; Department of Biomedical Genetics, University of Rochester Medical Center, 601 Elmwood Ave., Box 633, Rochester, NY 14642 USA; The Center for Visual Sciences, University of Rochester Medical Center, 274 Meliora Hall, RC Box 270270, Rochester, NY 14627 USA

**Keywords:** Secondary degeneration, Neuroinflammation, TNFα, Retinal ganglion cell, Macroglia

## Abstract

**Background:**

Glaucoma is an optic neuropathy that is characterized by the loss of retinal ganglion cells (RGCs) initiated by damage to axons in the optic nerve. The degeneration and death of RGCs has been thought to occur in two waves. The first is axogenic, caused by direct insult to the axon. The second is somatic, and is thought to be caused by the production of inflammatory cytokines from the activated retinal innate immune cells. One of the cytokines consistently linked to glaucoma and RGC damage has been TNFα. Despite strong evidence implicating this protein in neurodegeneration, a direct injection of TNFα does not mimic the rapid loss of RGCs observed after acute optic nerve trauma or exposure to excitotoxins. This suggests that our understanding of TNFα signaling is incomplete.

**Methods:**

RGC death was induced by optic nerve crush in mice. The role of TNFα in this process was examined by quantitative PCR of *Tnfα* gene expression, and quantification of cell loss in *Tnfα*^−/−^ mice or in wild-type animals receiving an intraocular injection of exongenous TNFα either before or after crush. Signaling pathways downstream of TNFα were examined by immunolabeling for JUN protein accumulation or activation of EGFP expression in NFκB reporter mice.

**Results:**

Optic nerve crush caused a modest increase in *Tnfα* gene expression, with kinetics similar to the activation of both macroglia and microglia. A pre-injection of TNFα attenuated ganglion cell loss after crush, while ganglion cell loss was more severe in *Tnfα*^*−/−*^ mice. Conversely, over the long term, a single exposure to TNFα induced extrinsic apoptosis in RGCs. Müller cells responded to exogenous TNFα by accumulating JUN and activating NFκB.

**Conclusion:**

Early after optic nerve crush, TNFα appears to have a protective role for RGCs, which may be mediated through Müller cells.

## Background

Optic neuropathies, of which glaucoma is the most common, are characterized by axonal degeneration in the optic nerve and apoptotic death of retinal ganglion cell (RGC) somas, leading to irreversible vision loss [1-3]. While the exact mechanisms that initiate RGC injury have not been clearly established, animal models of elevated intraocular pressure, axotomy, and optic nerve crush mimic the apoptotic pathways observed during glaucomatous neurodegeneration [4-9]. Although direct axonal injury ultimately leads to RGC somatic death, resident innate immune cells have long been suspected of playing a critical role during glaucoma [10-15]. Dendritic cells have been shown to infiltrate the damaged retina after crush injury [16]; however, it is the retinal glial cells, specifically macroglia (astrocytes and Müller cells) and microglia that comprise the principal populations of resident immune cells in the retina. Under normal circumstances these cells maintain retinal health, but after an injury they undergo an activation response to behave as innate immune cells by presenting antigens and releasing cytokines and other small molecules into the retinal tissue [17-19]. These signals initiate damage repair and remove critically injured neurons [15,20]; however, the effect of prolonged glial activation on RGC survival continues to be debated. Some research suggests that the innate immune response is critical for RGC protection after injury [21,22], while research in stroke and ischemia models demonstrate greater neuronal loss from activated glia [14,23,24]. More specifically in the latter paradigm, glial activation is thought to cause a second wave of RGC loss, termed secondary degeneration [7,11,25].

The model of secondary degeneration proposes that ganglion cell death during glaucoma occurs in two waves: first, that axonal injury culminates in the death of a subset of RGCs and the activation of retinal glia; and second, that the activated glia then produce cytotoxic molecules, such as inflammatory cytokines, that critically damage surviving RGCs [7,11,25-27]. It has been hypothesized that these cytokines are generated from either macroglia, principally Müller cells [28], or microglia [20], or both. Supporting evidence for this model comes from studies showing that minocycline, a broad spectrum anti-inflammatory drug, protects RGCs against optic nerve axotomy, experimental glaucoma, and optic nerve crush [7,29-31], implicating a damaging role for the immune response after injury. While many inflammatory cytokines have been linked to RGC degeneration [21,32,33], TNFα has been consistently associated with glaucomatous neuropathy [10,11,23,34-37].

TNFα is a pro-inflammatory cytokine that is elevated in several neurological diseases including multiple sclerosis [33], Alzheimer’s disease [38] and ischemia [24]. It is generated in the retinas of human glaucoma patients [35] as well as animal models of retinal injury [11,23,28,36,39-42]. Additionally, the receptors through which TNFα signals, TNFα receptor 1 (TNFR1) and TNFα receptor 2 (TNFR2), are also upregulated after retinal injury [35,43,44]. Isolating TNFα from the complex degenerative signaling pathways activated by RGC injury has yielded conflicting results about the role of this cytokine in RGC damage. TNFα is thought to contribute to RGC pathology following NMDA injection and optic nerve crush, which respectively cause RGC death within hours to days [5,7,45]; yet an intraocular injection of TNFα requires 2 weeks to cause axonal injury and 8 weeks before RGC somatic loss is significant [10,36,46]. Although TNFα injection does ultimately result in RGC loss, the disconnect in the timing of RGC damage suggests that TNFα may not simply flip a switch initiating degeneration, but may instead trigger a cascade of signaling networks that indirectly culminate in neuronal damage over time.

A possible explanation for this disconnect may be the opposing roles for TNFR1 and TNFR2 [43,47,48]. In human glaucoma, TNFR1 has been linked with the upregulation of pro-apoptotic proteins including BAX and CASP1 [37], and TNFR1 deficiency protected neuronal cell cultures from glutamate excitotoxicity [47], and increased RGC survival in a mouse model of optic nerve crush [11,37]. Conversely, TNFR2 deficiency increased neuronal susceptibility to glutamate [47], and caused greater RGC loss in a mouse model of ischemia/reperfusion [43]. Given that TNFα appears to play an important role during retinal injury, there is a clear need to better understand through which pathway(s) this cytokine is signaling. The present study investigates further the role of TNFα in the pathology of RGCs after optic nerve damage in mice. After optic nerve crush we detected a modest increase in *Tnfα* gene expression. Experimental evidence suggests that this inflammatory cytokine may have a protective role early in the RGC death process.

## Materials and methods

### Animals

Adult C57BL/6J mice (Jackson Laboratory, Bar Harbor, ME, USA) were handled in accordance with the Association for Research in Vision and Ophthalmology statement on the use of animals in research. All experimental protocols and the ethical care of the mice were reviewed and approved by the Institutional Animal Care and Use Committee of the University of Wisconsin. Mice were housed in microisolator cages and kept on a 12-hour light/dark cycle and maintained on a 4% fat diet (8604 M/R; Harland Teklad, Madison, WI, USA). *Bax-*deficient mice were generated from breeding *Bax*^*+/−*^ animals on a C57BL/6J background. *Tnfα*^*−/−*^ mice were obtained from the Jackson Laboratory and as a gift from Dr Matyas Sandor at the Univeristy of Wisconsin. NFκB expression was monitored with *cis*-NFκB^EGFP^ reporter mice [49] that were obtained from Dr Christian Jobin at the University of North Carolina. All genotypes were on the C57BL/6J background.

### Optic nerve crush surgery

Prior to surgery, mice were anesthetized with ketamine (120 mg/kg) and xylazine (11.3 mg/kg) and the eye numbed with a drop of 0.5% proparacaine hydrochloride (Akorn, Lake Forest, IL, USA). Optic nerve crush surgery was performed as previously described [5,9]. Briefly, a lateral canthotomy was performed followed by an incision through the conjunctiva at the limbal junction. The sclera was cleared of excess tissue before the optic nerve was exposed using self-closing N7 forceps (Fine Science Tools, Foster City, CA, USA), and clamped for 3 seconds. After surgery, the eye was covered with triple antibiotic ointment, and a subcutaneous injection of buprenex (0.2 mg/kg) was delivered to alleviate pain. Surgery was not performed on the right eye of each mouse, as previous studies have shown that mock surgery does not affect ganglion cell morphology or number [50,51].

### Intraocular injections

Mice were anesthetized with ketamine/xylazine and a drop of proparacaine was applied to numb the eye. A small hole was made through the conjunctiva and scleral tissue with a 30G needle, and then a 30G beveled Nanofil needle attached to a Nanofil syringe (World Precision Instruments, Inc., Sarasota, FL, USA) was inserted through the hole and a 2 μl volume of either 50 ng or 100 ng TNFα (Sigma, St Louis, MO, USA) was slowly delivered to the vitreous over 60 seconds. Care was taken not to damage the lens. After delivery, the needle was held in the eye for an additional 30 seconds before being retracted. A subcutaneous injection of buprenex was delivered to alleviate pain and the mouse was allowed to recover.

### RNA isolation and quantitative analysis of mRNA expression by quantitative PCR

Mice were euthanized with a lethal overdose of pentobarbital sodium prior to tissue harvest. Retinal tissue was collected and flash frozen on dry ice. At least three retinas were analyzed for each condition tested. Total RNA was isolated from the tissue using a solution of 50% phenol containing 1.67 M guanidine thiocyanate, 14.3 mM sodium acetate, 10.4 mM sodium citrate, 0.3% β-mercaptoethanol, and 0.005% Sarkosyl. Retinal tissue was sonicated in 1 ml of the phenol solution with 10 pulses at 50% power using a Branson Sonifier SLPe Energy Cell Disruptor (All-Spec Industries, Willmington, NC, USA). The RNA was then extracted with chloroform and precipitated with isopropanol. The pellet was washed in 70% ethanol and dried before being resuspended in DEPC-treated water (Fisher Scientific, Waltham, MA, USA). The total RNA concentration was determined using a BioPhotometer (Eppendorf, Hamburg, Germany). A DNase treatment with DNase I (Promega, Madison, WI, USA) was then performed on 4 μg of RNA to eliminate contaminating genomic DNA. The DNase-treated RNA samples were extracted with phenol and chloroform, and precipitated with ethanol. The pellet was washed with 70% ethanol and dried before being resuspended in DEPC-treated water (Fisher Scientific). Finally the RNA was converted to cDNA with oligo(dT) 15 primers and Moloney murine leukemia virus reverse transcriptase (Promega).

The cDNA samples were then diluted and 100 ng was analyzed by quantitative PCR (qPCR) for changes in gene expression of *Aif1*, *Gfap, Nrn1*, *Sncg*, *Tnfα* and *S16* ribosomal protein mRNA. The cDNA was added to diluted SYBR Green PCR master mix (Applied Biosystems, Grand Island, NY, USA) with 0.25 μM of each primer in a 20 μl reaction volume. Each cDNA sample was run in triplicate on an ABI 7300 Real Time PCR system (Applied Biosystems), superimposed on a standard curve to determine absolute transcript quantities, and normalized to *S16*. Cycling conditions were 95°C (15 seconds) and 60°C (60 seconds) for 40 cycles with a dissociation step. Primer sequences are listed in Table 1.

**Table 1:** **Quantitative PCR primer sequences**

**Gene name**	**Primer sequence 5’ → 3’**	**Size (bp)**
*Aif1*	Forward: AGAGAGGTGTCCAGTGGC	200
	Reverse: CCCCACCGTGTGACCTCC	
*Gfap*	Forward: CAAACTGGCTGATGTCTACC	269
	Reverse: AGAACTGGATCTCCTCATCC	
*Nrn1*	Forward: TTCACTGATCCTCGCGGTGC	238
	Reverse: TACTTTCGCCCCTTCCTGGC	
*Sncg*	Forward: GACCAAGCAGGGAGTAACGG	240
	Reverse: TCCAAGTCCTCCTTGCGCAC	
*Tnfα*	Forward: CGCGACGTGGAACTGGCAGAA	276
	Reverse: GTGGTTTGCTACGACGTGGGCT	
*S16*	Forward: CACTGCAAACGGGGAAATGG	198
	Reverse: TGAGATGGACTGTCGGATGG	

### Cell counts from retinal whole mounts

After euthanasia the superior portion of the eye was marked with a cautery, and then the whole eye was enucleated and fixed in 4% paraformaldehyde. After 50 minutes, the eye was rinsed in PBS and the anterior segment removed to create an eye cup. The retina was removed from the eye cup and placed with the ganglion cell layer (GCL) facing up onto a Superfrost Plus slide (Fisher Scientific); three additional relaxing cuts were made to allow the retina to lay flat. The whole mounts were stained with 300 ng/ml 4’,6-diamidino-2-phenylindole (DAPI; Fisher Scientific) and then thoroughly rinsed in PBS before being covered with Immu-mount (Fisher Scientific), coverslipped and stored at 4°C in the dark. Images were captured at 400× from all around the periphery of the retina, and nuclear counts were obtained from 24 distinct fields (120 μm^2^) for each retina and averaged together. Only rounded nuclei with at least one nucleolus, typical of both RGCs and amacrine cells in this layer, were included in the counts. Endothelial cells exhibiting elongated nuclei and no nucleolus, and densely staining astrocytes were excluded [52]. The GCL cell counts for each experimental retina were compared to the cell counts for the corresponding contralateral retina using the following formula to yield a percent change: [(cell count experimental) – (cell count control)]/(cell count control) × 100. Retinal ganglion cells represent about 50% of the GCL population [53]. It should be noted that while *Bax*^−/−^ mice have twice as many neurons as wild-type mice, the RGCs still represent about 50% of the GCL population [54].

### Immunofluorescent labeling

Whole eyes were fixed in 4% paraformaldehyde before the anterior segment was removed to create an eye cup. The eye cups were then rinsed in PBS, post-fixed overnight in 0.4% paraformaldehyde, and equilibrated in 30% sucrose in PBS. The eye cups were embedded in optimal cutting temperature compound (Fisher Scientific) in blocks and frozen on dry ice. Frozen sections were cut at 10 to 14 μm. Slides were rinsed in PBS and then blocked in 0.2% Triton-X, 1% BSA, and 5% donkey serum in PBS for 1 hour at room temperature. Primary antibodies (see Table 2) were incubated overnight at 4°C in PBS containing 1% BSA. Slides were thoroughly rinsed in PBS and incubated in Texas Red-conjugated or FITC-conjugated secondary antibodies (Jackson ImmunoResearch, Inc., West Grove, PA, USA) in the dark for 2 hours at room temperature in PBS containing 1% BSA. Slides were thoroughly rinsed in PBS before being incubated with 300 ng/ml DAPI for 5 minutes at room temperature. Finally, the slides were rinsed in PBS and coverslipped with Immu-Mount and stored at 4°C in the dark.

**Table 2:** **Primary antibodies**

**Protein name**	**Acronym**	**Species**	**Dilution**	**Company**	**Catalog #**
Allograft inflammatory factor 1	AIF1	Polyclonal rabbit	1:1000	WAKO^a^	019-19741
BRN3A	BRN3A	Monoclonal mouse	1:50	Millipore^b^	MAB1585
Caspase 3	CASP3	Polyclonal rabbit	1:1000	R&D^c^	AF835
Glial fibrillary acidic protein	GFAP	Polyclonal rabbit	1:1000	DAKO^d^	Z0334
JUN	JUN	Polyclonal rabbit	1:1000	Abcam^e^	Ab40766
Transcription factor SOX-9	SOX9	Polyclonal rabbit	1:1000	Millipore	AB5535
Tumor necrosis factor alpha	TNFα	Polyclonal goat	1:100	R&D	AF-410-NA

^a^Richmond, VA, USA; ^b^Billerica, MA, USA; ^c^Minneapolis, MN, USA; ^d^Carpinteria, CA, USA; ^e^Cambridge, MA, USA.

Whole mounts labeled with BRN3A were stained as previously described by Nadal-Nicolas and colleagues [55], with minor modifications. Briefly, following fixation of the globe, the anterior segment was removed and the eye cups were incubated in PBS containing 0.5% Triton-X100 and 2% donkey serum (Jackson ImmunoResearch, Inc.) for 1.5 hours at room temperature. They were then transferred into the same buffer containing primary antibody (see Table 2) overnight at 4°C. After incubation, the eye cups were thoroughly rinsed in PBS with 0.5% Triton-X100, and then fixed for an additional 10 minutes in 4% paraformaldehyde. Eye cups were rinsed in PBS and whole mounted onto Fisher Plus slides, and then incubated in 2% Triton-X100 and 2% donkey serum with 1:500 secondary antibody (Jackson ImmunoResearch) for 2 hours at room temperature. The whole mounts were rinsed in PBS and stained with 300 ng/ml DAPI for 5 minutes at room temperature. After a final wash with PBS, the slides were coverslipped with Immu-Mount and photographed.

### Microscopy

All immunofluorescent photographs were acquired using a Zeiss Axioplan 2 Imaging microscope (Carl Zeiss Microimaging, Inc., Thornwood, NY, USA) with a digital black and white camera. Images were analyzed using the Zeiss Axiovision Image Analysis software v4.6 (Carl Zeiss Microimaging, Inc.).

### Statistical analyses

Means from qPCR quantification are reported with the standard deviation of the mean, and cell counts are reported with standard error. Statistical significance between two means was determined using a two-sided Student’s *t*-test. *P* values were considered significant at a value equal to or less than 0.05.

## Results

### TNFα expression is stimulated following optic nerve crush injury

**Figure 1:**
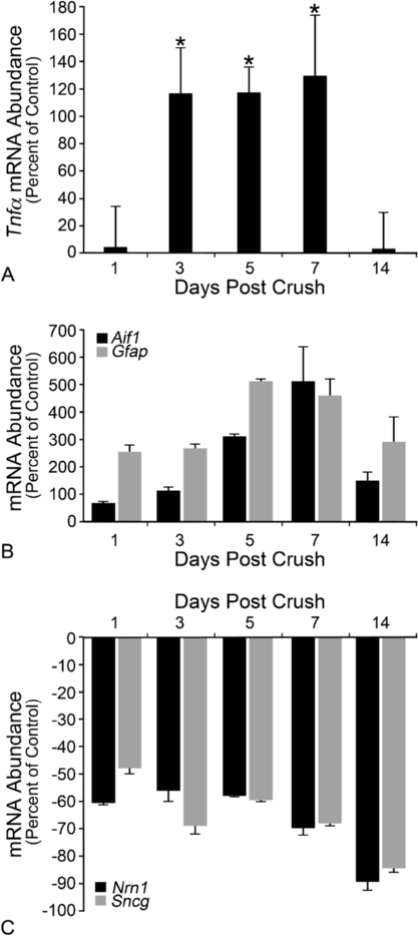
**Retinal** ***Tnfα*** **mRNA expression increases after optic nerve crush. (A)** *Tnfα* gene expression was monitored by quantitative PCR at 1, 3, 5, 7 and 14 days after optic nerve crush. Within 1 day of optic nerve crush, the level of TNFα expression began to increase compared to contralateral naïve eyes. At 3, 5, and 7 days after crush, TNFα expression was significantly elevated (*P* <0.05). By 14 days after injury, a time when about half of retinal ganglion cells have died from crush injury, TNFα levels were no longer statistically significant (*P* = 0.94). **(B)** The rise in *Tnfα* mRNA correlated with the increase in mRNA accumulation of glial activation markers *Aif1* (microglia) and *Gfap* (macroglia). Expression peaked for both markers between 5 and 7 days before declining at 14 days. The increase in expression in the injured retina was significant for all genes at all time points (*P* <0.01, relative to the contralateral eye). **(C)** Retinal ganglion cell gene markers, *Nrn1* and *Sncg*, declined within 1 day of optic nerve crush and remained low, consistent with RGC injury following optic nerve damage (*P* <0.05, relative to the contralateral eye). Data are presented as mean ± SE; n ≥3 for all timepoints.

An increase in *Tnfα* mRNA expression has been correlated with RGC and optic nerve injury; however, the time course of this expression to the best of our knowledge has not been documented. Therefore, the changes in *Tnfα* mRNA were analyzed by qPCR at 1, 3, 5, 7, and 14 days after optic nerve crush. Absolute mRNA levels in the retina were low across all time points in the injured retina. By 3 days after optic nerve injury, TNFα expression was significantly elevated in the injured retina compared to the contralateral eye, and remained significantly higher at 5 and 7 days after injury (Figure 1A; *P* <0.05). Fourteen days after injury, the difference in TNFα mRNA levels was no longer significant (Figure 1A; *P* =0.94). This pattern of expression mirrored the increase in markers for glial activation, specifically *Aif1* expression in microglia and *Gfap* expression in macroglia (Figure 1B). Conversely, transcripts of two genes selectively expressed in RGCs were downregulated during this time frame (*P* <0.05 for all genes and time points, crush relative to contralateral naïve eyes, Figure 1C), consistent with previous observations indicative of RGC damage from crush injury [56-59]. Western blots and enzyme-linked immunosorbent assay data did not reveal a quantifiable change in TNFα protein (data not shown).

### TNFα mediates retinal ganglion cell death through extrinsic apoptosis

An intraocular injection of TNFα induces RGC death and optic nerve degeneration [10,36,46], indicating that these cells are responsive to the toxic effects of this cytokine. Some discrepancies exist, however, in the mode of action by TNFα. First, toxicity to the RGC somas only manifests after several months from the initial exposure [36,46], making it unclear how TNFα participates in pathologies like *N*-methyl-d-aspartate injection and optic nerve damage, which lead to RGC death more rapidly [5]. Second, RGC death induced by axonal damage is absolutely dependent on the intrinsic apoptotic pathway, since these cells are completely refractory to this insult when lacking the pro-apoptotic *Bax* gene [54,60,61]. Conversely, the predicted mode of action for TNFα is through the extrinsic pathway, independent of BAX activation [15]. To test which apoptotic pathway is essential for TNFα-induced RGC death, wild-type and *Bax*^−/−^ mice were given an intraocular injection of TNFα and then assayed for cell loss at 6 and 8 weeks after injection.

**Figure 2:**
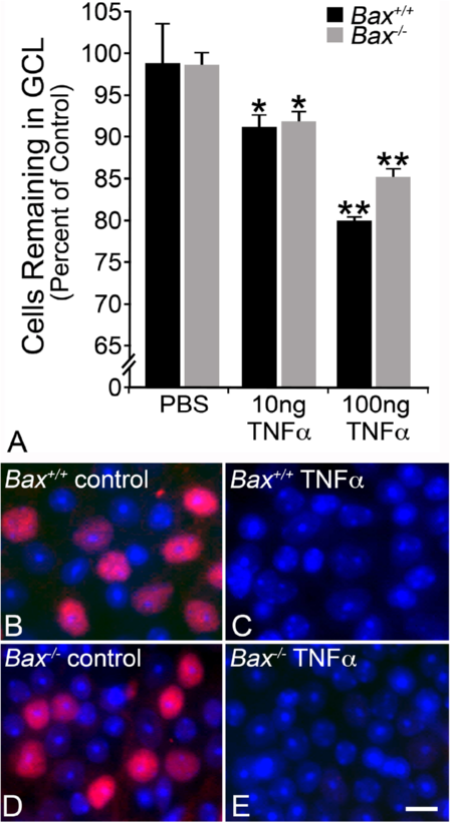
**TNFα mediates extrinsic apoptosis of retinal ganglion cells in a dose-dependent manner. (A)** A single intraocular injection of TNFα was delivered and the total number of cells in the ganglion cell layer (GCL) was obtained from 4’,6-diamidino-2-phenylindole-stained retinal whole mounts after 8 weeks. No cell loss was detected at 6 weeks (data not shown). Wild-type (black bars) and *Bax* ^−/−^ mice (gray bars) exhibited dose-dependent loss of total cells, indicating cell death occurred via a *Bax*-independent mechanism. The percent change was calculated by comparing the injected eye to the control eye. Statistics were calculated relative to the PBS-injected control group and are presented as mean ± SE (*P <0.005, **P <0.001); n ≥3 for all genotypes and conditions. **(B-E)** Retinal whole mounts were also stained for the retinal ganglion cell-specific transcription factor BRN3A. Approximately 40% of the neurons in the GCL stain for BRN3A in control retinas of both wild-type **(B)** and *Bax* ^−/−^ **(D)** mice [53,55]. Retinas exposed to a single injection of TNFα, 8 weeks prior, exhibit a complete absence of BRN3A staining (wild-type mice in **C**, and *Bax*-deficient mice in **E**). Scale bar =15 μm.

Consistent with other studies, injected TNFα induced cell loss, but only after 8 weeks (Figure 2A). The total number of cells in wild-type mice significantly declined 8 weeks after treatment with 10 ng and 100 ng TNFα (8.97% and 19.41%, respectively; *P* <0.005, compared to PBS-injected eyes). Similarly, *Bax*-deficient RGCs were also susceptible to 10 ng and 100 ng TNFα, with the total number of RGC layer neurons declining by 8.11% and 15.03% (*P* <0.001, compared to PBS-injected eyes). PBS injections alone did not cause significant cell loss (wild types *P* =0.36, knockouts *P* =0.40, relative to contralateral eye).

The effect of exogenous TNFα was not limited to a small percentage of RGCs, however. In similar experiments we stained retinal whole mounts for the RGC marker BRN3A, which can be depleted in damaged RGCs well in advance of cell death [56,62]. At 8 weeks after injection of 100 ng TNFα, there was undetectable BRN3A staining in retinas of both wild-type and *Bax*^−/−^ mice exposed to this cytokine (Figure 2B-E). Thus, even though there is only a loss of 30 to 40% of the RGCs (after correction of the percentage of RGCs that make up the total number of neurons in the ganglion cell layer [53]), exogenous TNFα appears to cause some level of damage to the entire population of these cells.

### TNFα deficiency exacerbates retinal ganglion cell injury after optic nerve crush

**Figure 3:**
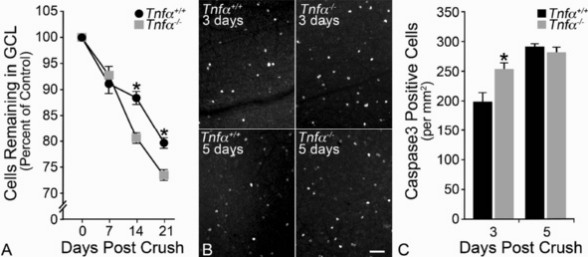
**Mice deficient for** ***Tnfα*** **have greater retinal ganglion cell loss from crush than wild-type mice. (A)** Wild-type and *Tnfα* ^*−/−*^ mice were subjected to optic nerve crush and analyzed for total retinal ganglion cell layer neuronal cells remaining at 7, 14, and 21 days after injury. The decline in cell numbers was not statistically significant between the two genotypes at 7 days, but at 14 and 21 days *Tnfα* ^*−/−*^ mice had significantly more cell loss (*P <0.001 for both time points). **(B,C)** Retinal whole mounts from wild-type and *Tnfα* ^*−/−*^ mice were analyzed for Caspase 3 (CASP3) activation 3 and 5 days after optic nerve crush. At 3 days, *Tnfα* ^*−/−*^ mice has a significantly greater number of CASP3+ cells (*P <0.05), but by 5 days the difference between the two genotypes was no longer significant. Results are presented at mean ± SE **(A,C)**; n ≥3 for all genotypes. Scale bar **(B)** =50 μm. GCL, ganglion cell layer.

To determine if TNFα contributes to RGC pathology after optic nerve crush, we performed surgery on *Tnfα*^−/−^ mice and obtained cell counts after 7, 14, and 21 days (Figure 3A). Although deletion of TNFR1 has previously been shown to confer significant resistance to acute optic nerve damage in mice [11], we observed greater cell loss in the *Tnfα*-deficient mice compared to the wild types. Although RGC loss was not significantly different 7 days after injury between the wild type and *Tnfα*^−/−^ mice (8.84% compared to 7.15% total cell loss, respectively; *P* =0.50), by 14 days *Tnfα*^−/−^ mice had lost 19.46% of the total cells, while the wild-type mice only showed an 11.61% decline in their total RGC layer neurons (*P* <0.001). Cell loss continued to decline in both genotypes 21 days after crush, but there was again significantly greater loss in *Tnfα*^−/−^ mice relative to the wild types (26.55% compared to 20.32%, respectively; *P* <0.001). In complimentary experiments, we also quantified the level of Caspase 3 (CASP3) activation after optic nerve crush in *Tnfα*^−/−^ mice. Previous studies have documented that CASP3 activity peaks between 3 and 5 days in this experimental paradigm [50,63]. Consistent with the cell count data, *Tnfα*^−/−^ mice exhibited significantly more CASP3 activity by 3 days after optic nerve crush compared to wild-type mice (*P* <0.05), although by 5 days the percent of CASP3+ cells was not statistically different (Figure 3B,C).

### A single injection of TNFα protects retinal ganglion cells when delivered prior to optic nerve crush

Dissecting out a single cytokine from a complex map of signaling pathways by genetic deletion is likely an over-simplification of any disease pathology. It is more plausible that TNFα is part of a larger orchestrated injury response, and without auxiliary injury signals this cytokine may be limited in its effectiveness. We therefore tested the effect of TNFα when delivered in conjunction with injury to RGCs by optic nerve crush. We tested two conditions: first, the cytokine was delivered prior to optic nerve crush to pre-activate the TNFα signaling network, which is expectedly enhanced following crush injury.

**Figure 4:**
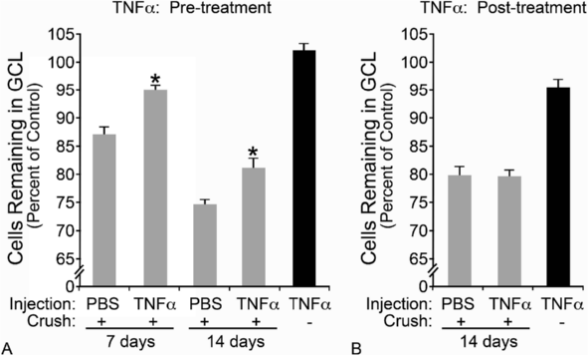
**Pre-treatment with TNFα improves retinal ganglion cell survival after crush.** Mice were treated with an intraocular injection of TNFα either **(A)** 5 days prior to optic nerve injury (pre-treatment) for neuronal cell counts 7 and 14 days after crush, or **(B)** 7 days after crush (post-treatment) and analyzed for cell counts 7 days later (14 days after crush injury). Pre-treatment with TNFα **(A)** reduced the amount of crush-induced cell loss by 50% 7 days after crush (*P* <0.001). Although cell loss continued to decline in both cohorts between 7 and 14 days after crush, the protection afforded by pre-treating with TNFα continued to be significant (*P* <0.001). Post-treatment **(B)** with TNFα did not affect RGC survival after crush (*P* >0.05). A single injection of TNFα alone yielded no significant cell loss after **(A)** 12 days, or **(B)** 7 days (*P* >0.05, injected eye relative to contralateral eye). Data are presented as mean ± SE; n ≥3. GCL, ganglion cell layer.

When an intraocular injection of 100 ng TNFα was delivered 5 days prior to crush injury, RGC loss was reduced by almost 50% 1 week after crush, compared to PBS-injected crushed mice (Figure 4A, *P* <0.001). This protective effect persisted, relative to PBS-injected eyes, to 14 days post-crush, although cell density decreased in both cohorts of mice (*P* <0.001). A second condition was also tested in which 100 ng TNFα was delivered 7 days after optic nerve crush; however, there was no statistically significant difference in cell survival between any of the groups subjected to crush (Figure 4B, *P* >0.05). These results support our findings above that TNFα may protect RGCs from optic nerve injury and, importantly, will not exacerbate damage when applied after the initial insult to the optic nerve.

### TNFα is not critical for glial activation by *Aif1* or *Gfap* monitoring

The data shown in Figures 3 and 4 illustrate a positive role for TNFα after RGC injury; however, the mechanisms of protection are still unclear. Others have reported that TNFα can act in an autocrine manner to augment the glial response after an injury, particularly in the astrocytes and microglia [64-67], which may help protect neurons from subsequent damage [48,68]. Therefore, in the absence of TNFα, the retinal glia may inadequately respond to an injury and render neurons more susceptible to degeneration. We next tested whether TNFα was critical for glial activation by comparing the microglial and macroglial activation responses after optic nerve crush in wild-type and *Tnfα*^−/−^ mice.

**Figure 5:**
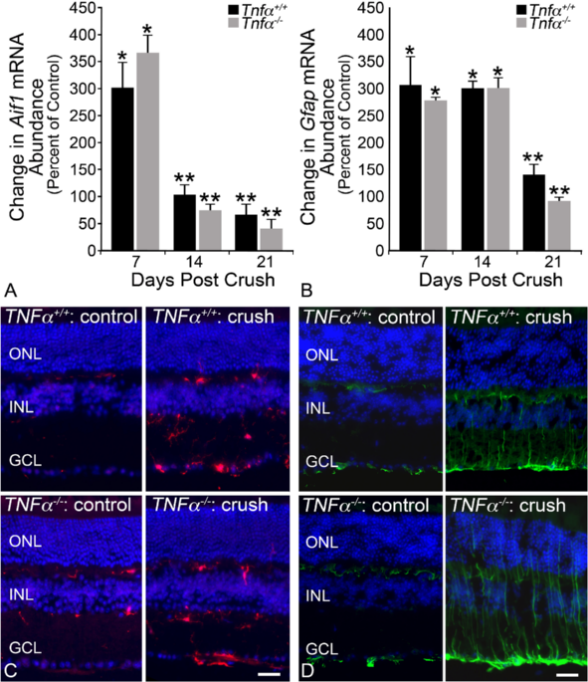
**TNFα is not required for glial activation as a function of** ***Aif1*** **or** ***Gfap*** **expression. (A,B)** Wild-type and *Tnfα* ^*−/−*^ mice were subjected to crush, and expression levels for activation markers of microglia (*Aif1*) and microglia (*Gfap*) were assessed by quantitative PCR. Wild-type and knockout mice followed similar glial activation trends for both markers. Micoglial activation **(A)** peaked in the injured retina 7 days after crush in both wild-type and *Tnfα* ^*−/−*^ mice. Macroglial activation **(B)** was also significantly elevated in the injured retina by 7 days in both genotypes, with peak expression occurring between 7 and 14 days. **(C,D)** Immunolabeling revealed a similar increase in allograft inflammatory factor (AIF)1 (red) and glial fibrillary acid protein (GFAP; green) protein 7 days after crush injury. AIF1-positive cells were prominent in the ganglion cell layer (GCL) and inner and outer plexiform layers. GFAP processes labeled through the retinal layers, consistent with Müller cell activation. *Tnfα* deficiency did not affect baseline levels of *Aif1* or *Gfap*. Overall, there was no discernible difference in the expression patterns of AIF1 or GFAP between the wild-type and *Tnfα*-deficient mice either before or after injury. Sections were counterstained with 4’,6-diamidino-2-phenylindole (blue). Data are presented as mean ± SD; *P <0.001, **P <0.01; n ≥3 for each genotype at each time point. Scale bar **(C,D)** =50 μm. INL, inner nuclear layer; ONL, outer nuclear layer.

The absence of TNFα minimally affected glial activation, as a function of *Aif1* and *Gfap* expression; wild-type and *Tnfα*^−/−^ mice showed almost identical activation trends. Microglial activation (*Aif1*) peaked in the injured retinas 7 days after crush (301% in wild-type mice, 366% in *Tnfα*^−/−^ mice, *P* <0.001 relative to contralateral eye) and then declined at 14 and 21 days to 66.6% and 41.9%, respectively (Figure 5A, *P* <0.05). The increase in *Aif1* expression was also detectable by immunofluorescence labeling in retinal sections, with positive cells manifesting in the inner and outer plexiform layers as well as the ganglion cell layer (Figure 5C). Macroglial activation (*Gfap*) in wild-type and *Tnfα*^−/−^ mice also trended similarly, with expression rising by 7 days to 305.6% and 277.0% in the wild types and *Tnfα*-deficient mice, respectively (Figure 5B, *P* <0.001 relative to contralateral eye). *Gfap* mRNA levels remained steadily elevated at 14 days after crush (301.7% in wild types, 299.9% in *Tnfα*^−/−^ mice), before considerably declining at 21 days to 139.2% and 91.56% (*P* <0.001 relative to contralateral eye). Glial fibrillary acid protein underwent a distinct morphological change with processes labeling through the retinal layers, consistent with Müller cell activation [20,30], and in the ganglion cell layer where astrocytes reside (Figure 5D). Overall, there was no significant distinction between microglial or macroglial activation in the wild-type and *Tnfα*^−/−^ mice.

### TNFα activates NFκB and causes JUN accumulation in Müller cells

Although TNFα does not appear to be required for glial activation as a function of *Aif1* or *Gfap* expression, it may be a critical extracellular signal that initiates protective networks within the activated glia after a retinal injury. Two known targets of TNFα signaling are the transcription factors NFκB [40,47] and JUN [69], and these two proteins have been shown to work cooperatively to regulate gene transcription [70-73]. The activation of NFκB has been correlated with resistance to ligand-mediated apoptosis [74,75], and JUN has been linked with both protective and apoptotic pathways in injured RGCs [51]. It is possible that TNFα is mediating protection through one or both of these transcription factors, so we next explored the effect of TNFα on NFκB activation and JUN accumulation.

**Figure 6:**
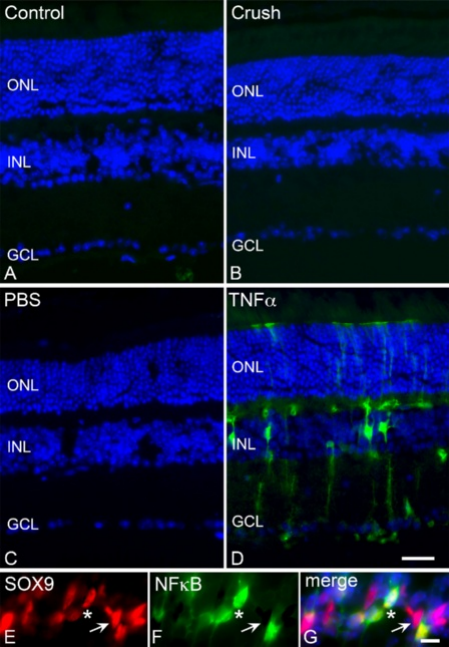
**An intraocular injection of TNFα activates NFκB in Müller cells.** *cis*-NFκB^EGFP^ reporter mice underwent optic nerve crush and were analyzed for enhanced green fluorescent protein (EGFP) expression as an indicator of NFκB activation. EGFP was not detected in control eyes **(A)**, nor was it detected at 1, 3, 5, 7, or 14 days after crush injury **(B)**; thus optic nerve injury and the resultant RGC death does not cause retinal NFκB activation. In contrast to this, while an intravitreal injection of PBS did not activate NFκB in the retina **(C)**, there was robust activation of NFκB as early as 1 day after an intravitreal injection of 100 ng TNFα **(D)**. EGFP (green) was present through the retina from the ganglion cell layer (GCL) to the outer limiting membrane, consistent with NFκB activation in Müller cells. **(E-G)** NFκB activity (EGFP expression) co-labeled with the Müller cell marker SOX9 (red, asterisk). NFκB was not expressed in cells absent for SOX9; however, some SOX9+ cells did not show activated NFκB (arrow). Sections were counterstained with 4’,6-diamidino-2-phenylindole (blue). Scale bars **(D)** =50 μm and **(G)** =10 μm; n ≥3 for each treatment at each time point. INL, inner nuclear layer; ONL, outer nuclear layer.

Using *cis*-NFκB^EGFP^ reporter mice [49], in which enhanced green fluorescent protein (EGFP) is transcribed by activated NFκB, we first examined whether optic nerve injury activated NFκB in the retina. We expected to find that NFκB activation would correlate with the time course of TNFα expression observed after crush; however, retinal sections did not reveal a detectable increase in NFκB activity following optic nerve crush (Figure 6B). We next tested the effect of an intraocular injection of TNFα into one eye of the reporter mice. Unlike crush, exogenous TNFα caused a clear activation of NFκB as early as 1 day after treatment (Figure 6D), and remained prevalent by 3 days before returning to baseline levels at 5 days (data not shown). The activity of NFκB was independent of the injection procedure, as eyes injected with PBS did not show any evidence of EGFP expression (Figure 6C). NFκB activation by TNFα co-localized with SOX9, which selectively labels Müller cells [76]. Interestingly, only a subset of Müller cells appeared to exhibit NFκB activation.

**Figure 7:**
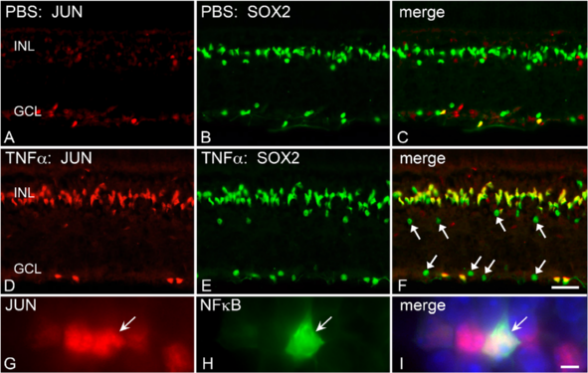
**An intraocular injection of TNFα causes nuclear accumulation of JUN in Müller cells. (A-C)** PBS injections revealed a baseline accumulation of JUN in the ganglion cell layer (GCL) with minimal activation in the inner nuclear layer (INL). **(D-F)** A single injection of TNFα resulted in widespread accumulation of JUN (red) in SOX2-positive Müller cells (green) as early as 7 hours after treatment. A subset of SOX2-positive cells were negative for JUN and exhibited rounded nuclei and no processes that may be amacrine cells (F, arrows). Additionally, in the GCL a subset of SOX2+ cells also exhibited JUN nuclear accumulation and may be astrocytes. **(G-I)** Most of the Müller cells responded to TNFα with an increase in labeling for JUN accumulation, but only a subset of these also upregulated NFκB (arrow). Cells were counterstained with 4’,6-diamidino-2-phenylindole (blue, I). After injection, sections were analyzed at 7 hours (n =3; A-F), and 24 hours (n =3; G-I). Scale bars (F) =50 μm and (I) =10 μm.

Optic nerve crush has previously been shown to cause nuclear accumulation of JUN in RGCs [50,51]. Similar to NFκB activation, however, an intraocular injection of TNFα caused a significant increase in JUN nuclear accumulation in the inner nuclear layer, and to a lesser extent in the GCL (Figure 7F). This accumulation occurred as early as 7 hours after TNFα exposure, and co-localized with SOX2, a marker for Müller glia and amacrine cells [77,78]. A morphologically distinct population of SOX2-positive cells in the GCL also showed an accumulation of JUN, which could be displaced amacrine cells or astrocytes (Figure 7F, arrows). While TNFα caused JUN accumulation in the majority of the Müller cell population, only a subset of the JUN-positive cells also exhibited NFκB activation.

## Discussion

The role of TNFα in neurodegeneration has been extensively studied, yet caveats still remain in understanding the mechanism by which it contributes to disease. In the context of RGC injury and death, TNFα has been considered by some researchers as detrimental and has been modeled as a secondary inducer of RGC loss [7,11,13,25-27,79]. In accordance with the literature supporting this theory, we found that *Tnfα* mRNA is elevated in our model of optic nerve crush, and that the cytokine does lead to a delayed loss of RGCs through an extrinsic apoptotic mechanism. However, a single intraocular injection of TNFα does not cause rapid RGC loss as is seen with other ocular injury models to which TNFα has been linked. Additional research supports that TNFα is beneficial and protective to neurons [43,47,48,80]. Consistent with this we found that genetic deletion of the *Tnfα* gene rendered mice more susceptible to optic nerve injury, and that pre-treatment with exogenous TNFα promoted RGC survival after crush. It appears contradictory that TNFα is both detrimental and protective to RGCs; however, our data present a potentially critical timing component that has not previously been studied in regards to TNFα signaling in the retina. More specifically, it appears that early TNFα exposure prior to an injury may be protective, while chronic TNFα expression may eventually culminate in neuronal damage and loss.

This phenomenon of neuronal protection from pre-conditioning has been previously observed in stroke patients, in which those with a history of transient ischemia attacks fared better following a cerebral infarction than those without a similar history [81]. This protection has been mimicked in cell culture models of neuronal insult and animal models of brain ischemia with TNFα exposure prior to a damaging stimulus [47,82]. Early treatment with TNFα has been positively correlated with survival pathways mediated by a number of proteins, including phosphoinositide-3-kinase [47], the transcription factor NFκB, and the histone acetyltransferase CREB binding protein [82]. The upregulation of CREB binding protein was observed only in neurons, even when co-cultured with astrocytes [82], suggesting that TNFα was acting directly on the neurons and not being mediated through the glia. However, in the retina, astrocytes are only one subpopulation of glial cells - Müller cells and microglia also contribute to retinal health and injury repair, complicating the mechanism by which TNFα may be promoting RGC survival.

TNFα signals through TNFR1 and TNFR2, and following retinal ischemia and a mouse model of glaucoma, both receptors are upregulated in cells of the inner nuclear layer and GCL [43,44]. TNFR1 is a transmembrane protein with an intracellular death domain that, upon activation, can interact with adaptor proteins and initiate apoptosis through CASP8 [37,83,84], and several studies have shown that *Tnfr1*^−/−^ mice exhibit significantly less RGC loss than wild-type mice after injury [11,43]. In comparison, TNFR2 does not contain a death domain and has been linked with sustained NFκB activation [43,47]. Unlike the RGC protection seen after injury with *Tnfr1* deficiency, *Tnfr2*^−/−^ mice fair worse than wild-type mice following ischemia [43] and glutamate excitotoxicity [47]. Interestingly, the *Tnfα*^−/−^ mice in our study more closely reflect the enhanced RGC pathology seen in *Tnfr2*^−/−^ mice after injury. This might suggest a preference for TNFα to bind TNFR2, possibly explaining the protection afforded when TNFR1 is genetically ablated, restricting TNFα to signal through the TNFR2 protective networks. Alternatively, *Tnfr2* deficiency may enhance apoptotic signals through TNFR1, rendering central nervous system tissue more susceptible to injury. In both scenarios, it seems critical to understand the proteins downstream of each receptor that are being affected after injury, and in particular which retinal cell types are responding to this cytokine.

It is important to note that while we have shown an early protective potential of TNFα, the long-term consequence of TNFα exposure still appears to be detrimental. This dual function of TNFα may reflect different responses of individual cell types to this cytokine, which is consistent with a recent publication by Dvoriantchikova and Ivanov [85]. Their research found that, in response to TNFα, RGC cultures exhibited sustained JNK activation and death, while astrocytes upregulated NFκB and promoted survival [85]. Therefore, the localization of TNFα expression and the cells responding to this cytokine will influence whether TNFα has a beneficial or detrimental effect on RGCs. TNFα is expressed by a number of innate immune responders, and has been co-localized to optic nerve head, nerve fiber layer, GCL, and the inner nuclear layer of human glaucoma patients [34,35,43,86], and is upregulated by macroglia and microglia in the optic nerve and optic nerve head [11,34]. Additional studies have shown that dendritic cells also infiltrate the retina following a similar optic nerve crush paradigm described here [16,87], and it is conceivable that they may be the source of TNFα. The proximity of TNFα production to specific cell types in the retina may generate the differential protective versus detrimental effects. The protective effects may occur through an indirect mechanism, by TNFα-induced changes in retinal glia. Based on the rapid induction of NFκB activity and JUN accumulation after exposure to exogenous TNFα, we attribute this rapid response to Müller cells (see below). Conversely, the detrimental effects may result from a direct interaction with the RGCs. Kitaoka and colleagues noted that intravitreal injection of TNFα in rabbits resulted in relatively early-onset axonal damage followed by soma death many weeks after TNFα exposure [40].

Unlike the delayed effect of TNFα on RGC somas, we have shown that Müller cells respond rapidly to TNFα within 1 day of exposure by accumulating JUN and upregulating NFκB, two known targets downstream of TNFα [85]. It is important to note that JUN is activated by phosphorylation; however, the p-JUN antibody is less reliable than that for JUN due to cross-reactivity [50]. Therefore, the data presented in this manuscript are documented as nuclear accumulation of JUN rather than activation. Additionally, JUN is known to autoregulate its own expression following its activation [88], and JUN levels have been used as a surrogate of JUN activity. In response to an intraocular delivery of TNFα, both JUN and NFκB exhibit nuclear activity, and it is interesting that the primary glial cells responding to TNFα are the Müller cells. However, while JUN accumulation was present in all of the Müller cells, only a subset exhibited NFκB activity. This might suggest that JUN is upstream of NFκB, and that all of the Müller cells have not yet been able to activate the latter gene, although the literature suggests a more complex interplay between these transcription factors [71,73]. It is also unclear if either of these pathways are involved in the protective effect of TNFα. However, given the strong association of NFκB with survival pathways [47,73], it is possible that the cells expressing this transcription factor might be mediating the protective effect seen in our studies involving intravitreal injection of exogenous TNFα. A paradox with this interpretation, however, is that neither JUN nor NFκB were activated after optic nerve crush. While this may have been a function of reduced or more localized levels of TNFα production (such as by infiltrating dendritic cells), it remains unclear how the endogenous TNFα signaling response provides a protective environment for RGCs. Further studies involving cell-specific ablation of one or both of these transcription factors are needed to decipher whether they play a role in the endogenous TNFα protective effect.

The signaling pathways activated in the injured retina are complex, but we have identified a critical timing component in TNFα signaling: specifically, that early exposure to this cytokine protects RGCs from subsequent optic nerve damage. A considerable amount of literature has identified a damaging role for TNFα, yet this research has indirectly focused on the effect of TNFα signaling late after injury. Future studies should consider the advantage of early immune activation in the retina, specifically with an emphasis on Müller cell activation. By bolstering protective pathways early, rather than eliminating cytokine signaling entirely, RGC loss may be minimized following a severe insult and improve the prognosis for patients with optic neuropathies, such as glaucoma.

## Conclusion

Our studies underscore that while long-term exposure to TNFα is toxic to RGCs, this cytokine appears to initiate protective pathways that improve RGC survival immediately following optic nerve injury. The mechanism of protection may be occurring through TNFα activation of Müller cells.

## Abbreviations

CASP: Caspase
DAPI: 4’,6-diamidino-2-phenylindole
EGFP: enhanced green fluorescent protein
GCL: ganglion cell layer
PBS: phosphate-buffered saline
PCR: polymerase chain reaction
qPCR: quantitative polymerase chain reaction
RGC: retinal ganglion cell
TNFα: tumor necrosis factor alpha
TNFR1: tumor necrosis factor alpha receptor 1
TNFR2: tumor necrosis factor alpha receptor 2
